# The relationship between changes in neighborhood physical environment and changes in physical activity among children: a prospective cohort study

**DOI:** 10.1186/s12966-023-01478-2

**Published:** 2023-07-07

**Authors:** Francesco Acciai, Robin S DeWeese, Kristen Lloyd, Michael J Yedidia, Michelle Kennedy, Katherine Isselmann DiSantis, David Tulloch, Punam Ohri-Vachaspati

**Affiliations:** 1 College of Health Solutions, Arizona State University, Phoenix, AZ USA; 2 Center for State Health Policy, Institute for Health, Health Care Policy and Aging Research, Rutgers University, New Brunswick, NJ USA; 3 Center for Tobacco Studies, Rutgers University, New Brunswick, NJ USA; 4 College of Population Health, Thomas Jefferson University, Philadelphia, PA USA; 5 Department of Landscape Architecture, Rutgers University, New Brunswick, NJ USA

**Keywords:** Physical activity, Physical activity environment, Physical activity upgrades, Community Assessment, Program Planning and evaluation

## Abstract

**Background:**

Physical activity (PA) is associated with positive health outcomes over the entire life course. Many community-based interventions that promote PA focus on implementing incremental changes to existing facilities and infrastructure. The objective of this study was to determine if such upgrades were associated with increases in children’s PA.

**Methods:**

Two cohorts of 3- to 15-year-old children (n = 599) living in 4 low-income New Jersey cities were followed during 2- to 5-year periods from 2009 to 2017. Data on children’s PA were collected at 2 time points (T1 and T2) from each cohort using telephone survey of parents; data on changes to existing PA facilities were collected yearly from 2009 to 2017 using Open Public Records Act requests, publicly available data sources, and interviews with key stakeholders. PA changes were categorized into six domains (PA facility, park, trail, complete street, sidewalk, or bike lane) and coded as new opportunity, renovated opportunity, or amenity. A scale variable capturing all street-related upgrades (complete street, sidewalk, and bike lane) was constructed. PA was measured as the number of days per week the child engaged in at least 60 min of PA. The association between change in PA between T1 and T2, ranging from − 7 to + 7, and changes to the PA environment was modeled using weighted linear regression controlling for PA at T1, child age, sex, race, as well as household and neighborhood demographic and socioeconomic characteristics.

**Results:**

While most measures of the changes to the PA environment were not associated with change in PA between T1 and T2, the street-related upgrades were positively associated with the change in PA; specifically, for each additional standard deviation in street upgrades within a 1-mile radius of their homes, the change in PA was 0.42 (95% CI: 0.02, 0.82; p = 0.039) additional days. This corresponds to an 11% increase over the mean baseline value (3.8 days).

**Conclusions:**

The current study supports funding of projects aimed at improving streets and sidewalks in cities, as it was shown that incremental improvements to the PA environment near children’s homes will likely result in increased PA among children.

## Introduction

Regular physical activity (PA) has multiple physical and mental health benefits for children, including improved bone health, weight status, cardiorespiratory and muscular fitness, cardiometabolic health, cognition, and reduced risk of depression. [1] However, only 24% of 6-17-year-old children engage in the recommended amount of at least 60 min of PA each day, [2] posing a significant public health concern. Acknowledging the need for interventions to combat childhood obesity, the Centers for Disease Control and Prevention (CDC) and the National Academy of Medicine have recommended community-driven changes in the PA environment that would increase the access to and appeal of places where people can be physically active and make it easier to integrate physical activity into daily life. [3, 4] Research in this area points to the association between environmental features and child physical activity levels. Cross-sectional studies have concluded that several aspects of the built environment, including bike lanes, high land-use mix, and residential density are associated with increased PA in children and adolescents. [5, 6] Prospective, predominantly single-site studies of targeted interventions and natural experiments show similar results. For example, in Denver, development of passive open space into a recreational park was linked to greater use of the space and increased energy expenditure by adolescent males. [7] Similarly, in San Francisco, substantial renovations in two parks, which included installing new playground and fitness equipment, landscaping, and building a recreation center, resulted in increased park use—measured by pre- and post-installation use—among both adults and children. [8].

Infrastructural elements of neighborhoods (e.g., street interconnectivity), sometimes defined as macroscale features, [9] are relatively permanent aspects of the urban landscape. Changes to these elements are generally difficult to implement because they require significant financial investments over an extended period of time. [10] Consequently, community-initiated interventions frequently focus on upgrades to existing PA opportunities that do not entail major infrastructural changes (e.g., adding basketball courts or ballfields to existing parks or playgrounds, resurfacing of playgrounds), as well as other microscale changes that improve safety and attractiveness of engaging in PA (e.g., renovated sidewalks, added signage, bike lanes). [9, 11–13] Recent studies have found that these upgrades may affect PA by expanding opportunities to engage in PA in the neighborhood as well as by increasing active transport in the daily routines of neighborhood residents. For example, Cain et al. found that several microscale elements (e.g., crosswalk amenities, curb quality, sidewalk slope) were associated with children actively commuting to their destinations and with engaging in PA in their neighborhoods. [14] Comparably, a study based in Montreal and Toronto reported that a higher summary microscale score was associated with higher odds of leisure walking among adults. [15].

Existing research typically uses publicly and commercially available data sources to document changes in the neighborhood PA environment, such as construction of a new park or opening and closing of indoor PA facilities (e.g., gyms, rec centers). [15–17] Virtual audits using Google Street View [18] and other virtual tools [9] have also been used for this purpose. Weaknesses in these approaches include (1) their limited capacity to identify upgrades to existing facilities, which are frequently the focus of interventions, (2) misclassification arising from reliance on data from sources compiled for non-research purposes, (3) lack of a standardized coding scheme for types and extent of changes, and (4) focusing on a single/few changes in the PA environment without controlling for other features of that environment. [19].

Using a longitudinal design and a comprehensive, detailed protocol to identify incremental changes to the PA environment, the current study examined the association between public PA upgrades and changes in children’s PA behaviors over time, while accounting for several other relevant aspects of the environment. This design affords a high degree of control and adds confidence to the inferences drawn from the current findings, as compared to the cross-sectional analyses that predominate among prior studies.

## Methods

### Study overview

The New Jersey Child Health Study (NJCHS) is a longitudinal study investigating the impact of food and PA environments on children’s weight status and associated behaviors in four low-income, racially/ethnically diverse US cities, located in the state of New Jersey: Camden, New Brunswick, Newark, and Trenton. Two randomly sampled cohorts of children were surveyed at two time points between 2009 and 2017, with the follow-up period ranging from 2 to 5 years. [20–23] This dataset was complemented with data on upgrades to the community PA environment that occurred in the four study cities during the follow-up period. The Rutgers and Arizona State University Institutional Review Boards approved the study protocol.

### Household survey – characteristics of children, their Locale, and physical activity

Between 2009 and 2017, survey data were collected at two time points for each of the two panels of households, for a total of 4 data collection occasions, each of which occurred simultaneously across the four study cities. Panel 1 (P1), Time 1 (T1) interviews were conducted in 2009-10. Data for this panel were collected using a random-digit-dial survey of households with landline telephones. Time 2 (T2) interviews for P1 were completed in 2014-15. Panel 2 (P2) used a multi-frame landline and cell phone sampling method. Cell phones were added to the sampling frame because of declining use of landline phones. T1 and T2 interviews for P2 were conducted in 2014 and 2016-17, respectively.

**Fig. 1:**
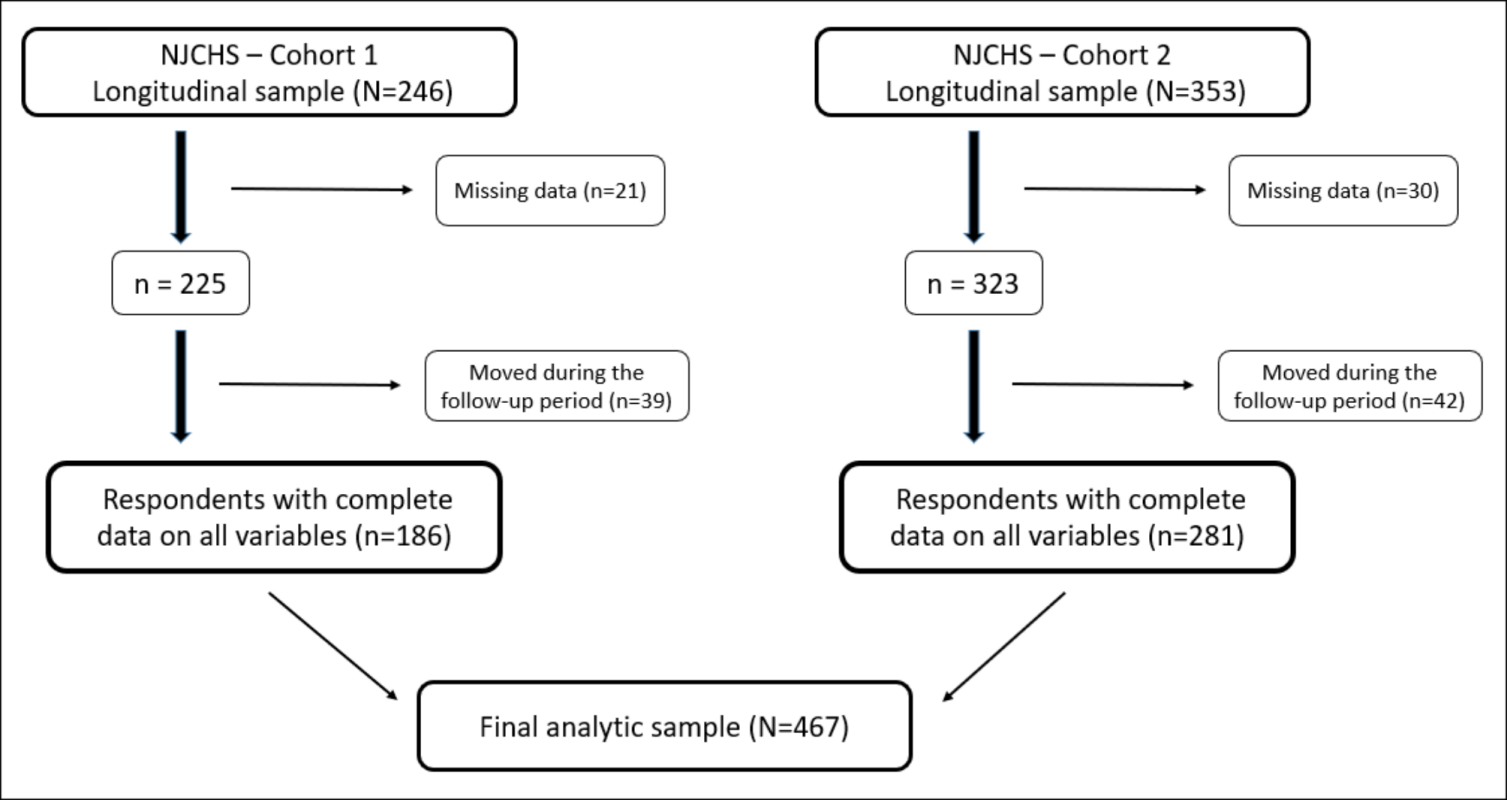
New Jersey Child Health Study (NJCHS): From the full longitudinal sample to the analytic sample, for each of the two cohorts

In both panels, households located within the study city and having at least one child between 3 and 15 years old were eligible for inclusion in the study. Computer-assisted telephone interviews were conducted in English or Spanish by trained research staff. The respondent for both panels was an adult, at least 18 years old, and primarily responsible for food purchasing decisions for the family; in over 94% of cases this was a parent or grandparent. Data were collected on the respondent and on one child (referred to as the index child). In households with multiple age-eligible children, a computer program randomly selected the index child. All households were eligible to participate in the follow-up interview, as long as they still resided in the study city. Interviews took on average 36 and 30 min to complete for panel 1 and panel 2, respectively. Across the two panels, response rates were 49% and 36% for T1 and T2, respectively. Survey questions were derived from previous research and included relevant individual and household characteristics at T1. Respondents were asked at both time points about their own and the index child’s height, weight, food and PA behaviors, household demographics and socioeconomic status, as well as home address. At T2, respondents were also asked if the child had lived at any other address, along with duration of residence, in the years between T1 and T2. Over 97% of the households had complete address information. Figure 1 provides details on sample size for the two panels. From the two panels combined, completed interviews at two time points were available for 599 households.

Regarding physical activity, respondents were instructed to think of the index child’s PA in the past 7 days, and were asked: *Adding up all the time the index child spent in any kind of physical activity that increased his/her heart rate and made him/her breathe hard, on how many days was s/he physically active for a total of at least 60 min per day?* This question was adapted from the CDC Youth Risk Behavior Surveillance System Survey, [24] and similar questions have been used, for instance, in the National Survey of Children’s Health [25] and in the California Health Interview Survey. [26] The PA outcome was measured as change in the number of days per week in which the child engaged in at least 60 min of PA. Because the range of the PA behavior variable was 0–7 at both time points, the measure of the change in PA between T1 and T2 ranged from − 7 to + 7.

### Exposure variable – changes in the community PA environment

We developed a comprehensive protocol for identifying and coding upgrades to existing PA environments which was informed by convening a panel of experts on children’s PA and by using public records based on actual project data, including budgets, scope of changes, locations, and dates of completion. These data are critical for capturing community PA environment changes. A sequence of data gathering strategies was pursued in each of the four study cities; (1) open public records act (OPRA) requests of city and county agencies for plans, contracts, site maps, and completion dates of relevant projects; (2) routine input from community organizations and key informants on planned and completed PA-related interventions; monitoring of print and social media reports; (3) on-site visits to confirm changes were completed and available for use as planned. This level of detail ensured that the vast majority of both macro and microscale changes in the neighborhood PA environment were tracked over time. [27].

Municipal and county agencies were used to identify upgrades in the PA environment. Requests through the New Jersey OPRA were submitted to each of these agencies in the four study cities to obtain information on infrastructure improvements occurring during the study period (2009–2017). A panel of experts with extensive experience examining food and PA environments advised the study team in identifying key elements of upgrades to the PA environment with the highest potential for impacting children’s weight status. Based on the experts’ advice, a systematic protocol was developed for collecting and coding information on PA upgrades. OPRA requests were made for municipalities and types of upgrades, which included, but were not limited to, sidewalks, streets, lighting, signage, parks, and public recreation facilities. Requests were directed to a comprehensive set of governmental agencies including city and county offices related to streets, parks and recreation, transportation and engineering, as well as state agencies including park commissions and natural lands, and water commissions. In response to requests, agencies provided engineering and construction maps, contracts, budgets, photographs, timelines, and other project specifications; local and city planners and relevant department personnel assisted with interpreting materials to ensure the following key data was extracted: street address or nearest intersection, city, date of completion, description of change, and information to apply domain and coding scheme (described below). Changes were geocoded for proximity to children’s residences. The date when the change took place was also obtained to establish when an opportunity became available for use (rather than when the project was funded or paid for) in order to calculate the duration of the children’s exposure to each of the monitored changes once they were implemented.

Media sources and community development organizations (CDO) were used to conduct cross-checks and confirm information obtained through OPRA requests. Public records can be difficult to interpret, often necessitating the use of key informants to clarify and validate information identified from government agencies. All data were double coded by a team of two trained research staff, and discrepancies were resolved through consultation with the principal investigators and key informants. Additionally, 10% of the sites where changes were documented were randomly selected and visited by field staff in order to validate data through visual inspection and/or consultation with key informants. These audits confirmed that the protocol accurately characterized changes.

### Coding scheme

Observed changes to the existing PA environment were first categorized into six domains: (1) public PA facilities, (2) parks, (3) trails, (4) bike lanes, (5) sidewalks, and (6) complete street elements. Upgrades to private PA facilities (e.g., gyms requiring paid memberships) were not tracked since these types of facilities were not accessible for free and were not prominent in the communities under study. Changes within each domain were coded as shown in Table 1:

**Table 1:** Physical activity domains and examples of changes documented and classified

Domain and Code	Examples
PA Facilities	
New Opportunity	New: pool, basketball court, playground equipment, soccer field
Renovated Opportunity	Resurfaced playground, replaced gym floor, resurfaced tennis court
Amenity	New/replaced: lighting, fencing, benches, bleachers, landscaping
Parks	
New Opportunity	New: basketball court, soccer field, fitness nodes, spray ground, pool, walking path, playground equipment, volleyball courts, artificial turf, indoor structure for pitchers and catchers, walking track, skate park, batting cages, tennis court
Renovated Opportunity	Resurfaced walking path, resurfaced basketball court, reset of basketball backboard, water access feature (kayak/canoe launch), refurbished playground equipment, resurfaced tennis courts
Amenity	Trash bins, benches, directional assistance, signage, bike rack, landscaping/gardens, tables and chairs, restrooms, safety services at fitness nodes, outdoor lighting, fencing, picnic area, outdoor shelter, new boathouse, performance space, curbs, field striping, open shade structure, new parking lot, game table
Trails	
New Opportunity	New trail or elongation
Renovated Opportunity	New access point
Amenity	Signage, trash bins, benches, directional assistance, bike rack, landscaping/gardens
Complete Streets	
Amenities	Upgraded intersections, striping, video detection, crosswalk signage, speed bumps, corner bump outs, rumble strips, signage, lane diets, new traffic signals, bumpy pads, repaved streets, landscaping, drainage, curb cuts, benches, trash bins, raised intersections, medians
Sidewalks	
New	New sidewalks
Renovated	Resurfaced/replaced sidewalks
Bike Lanes	
New	New bike lanes
Renovated	Re-painted bike lane

- New opportunity – a new opportunity within an existing facility (e.g., a new basketball court in a park).
- Renovated opportunity – the renovation of an existing opportunity within an existing facility (e.g., resurfacing of a playground, replaced gym floor).
- Amenity – features that may not directly impact PA but may attract more users to the site and support use of PA opportunities (e.g., lighting, trash bins, or benches in a park). Amenities are likely to contribute to pleasantness or enhance security, thus indirectly promoting use of the PA opportunity. [14, 22, 23].

Table 1 describes the coding scheme for changes to the PA environment and provides examples for each of the six domains. For PA facilities and parks, each type of new opportunity was coded separately; for example, if a new playground and a new basketball court were constructed within a park they would be coded as two new opportunities within the park. However, multiple new opportunities of the same type within the same facility – for example two tennis courts within a park – were counted as only one new opportunity. The same approach was applied to renovated opportunities.

Each of the study cities has an extensive network of sidewalks. In order to obtain information on changes and improvements to sidewalks, project-specific construction maps acquired primarily through OPRA requests were reviewed. Information on bike lanes was similarly acquired. In addition, CDOs promoting and implementing the upgrades shared project plans prior to completion. Descriptions of each upgrade’s locations were converted into geospatial data representing the upgrade footprints, and then converted to potential access points at all intersections along the length or border of the upgrade.

### Calculating exposure

**Fig. 2:**
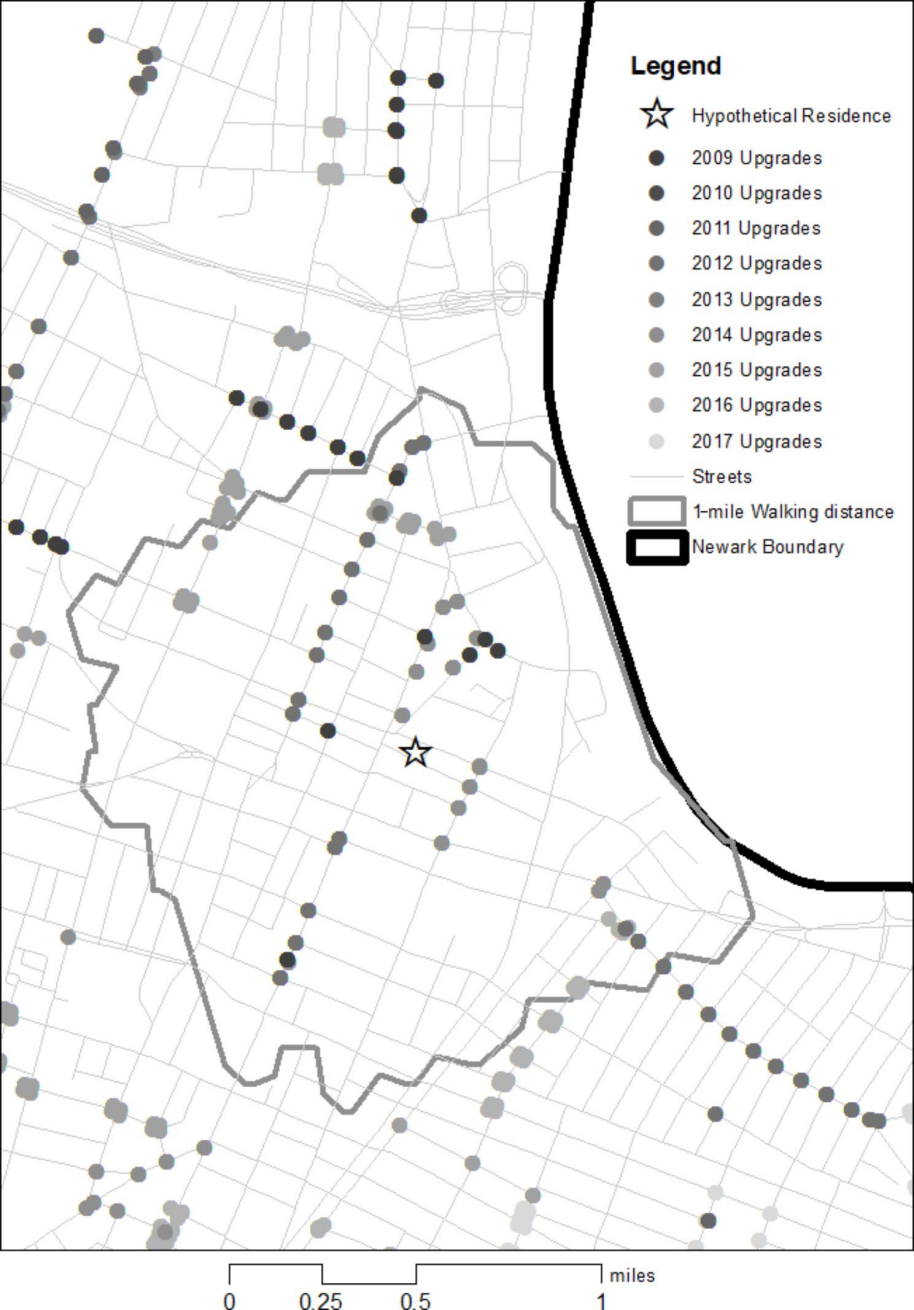
Hypothetical respondent’s place of residence, along with the measurement of PA upgrades over time within the 1-mile road network buffer

The exposure variables are longitudinal measures of PA upgrades that occurred within the environment around each child’s home, specifically, within a 1-mile radius. To calculate this, all home address information collected in the household surveys was geocoded. Based on survey dates and respondents’ reported duration of residency at a given address, a longitudinal address database was created assigning each household a location for every month elapsed between T1 and T2. The proximity from each geocoded address to each geocoded environmental upgrade was then calculated, with the universe of upgrades for each panel including only those with effective dates occurring after that panel’s first T1 interview. The proximity was the shortest, walkable road network distance from the respondent’s home to each PA upgrade within the city and the 1-mile buffer around the city measured using the OD (origin-destination) Cost Matrix analysis tool in the Network Analysis extension of the Geographical Information System ArcMap versions 10.1–10.8. [28] Fig. 2 illustrates an example of an hypothetical survey respondent’s place of residence, along with the measurement of PA upgrades over time within the 1-mile buffer.

Next, the number (i.e., counts) of each type of change in the PA environment within a 1-mile radius around a child’s home at T1 and T2, as well as for each month in between, was calculated. Lastly, the difference between the average of monthly counts over the 18 months preceding the T2 interview and the count value at the time of the T1 interview was calculated. Using a monthly average of the period preceding T2 allowed for accounting for not only the changes that occurred between T1 and T2 in the exposure measure, but also the timing and duration of these changes. After examining the distribution in this exposure variable across the three different radii (0.25, 0.5, and 1 mile(s)), we found the 1-mile radius to exhibit the most variation and to be best suited for analysis.

22% (n = 120) of the complete-case households in the longitudinal sample moved between their T1 and T2 interview. Implicit in the conceptualization of the exposure is that PA upgrades that occur between T1 and T2 are enhancements to the T1 environment—the base environment that the child experienced prior to the first interview. When a child moved between T1 and T2, the exposure variable as calculated would no longer capture the change in these incremental enhancements since the upgrade counts pertain to a different base PA environment. Nevertheless, of the 120 children who moved between T1 and T2, we retained in the sample 39 who (1) moved 6 months or less before their T2 interview or (2) moved less than 0.5 miles from their T1 address. In these cases, the PA upgrades children were exposed to were mostly experienced in the T1 environment (either because they moved right before T2 or because they moved nearby).

### Demographic and contextual factors

Children’s age, sex, and race/ethnicity, as well as household income were collected from parents in the household survey. Based on race/ethnicity, children were classified as Hispanic, non-Hispanic Black, non-Hispanic White, and other. Because of the small sample size, the last two groups were combined in the analysis. Household income was calculated as the ratio of the household income to the federal poverty line (FPL) for the year in which the survey was conducted. Other control variables included the number of months elapsed between T1 and T2, and two contextual-level variables, the median household income and total population at the block group level, both publicly available in the American Community Survey 5-year estimates, corresponding with the year of the survey. [29].

### Statistical analysis

To model the change in PA (i.e., the change in the number of days per week in which the child exercised for at least 60 min) between T1 and T2, we used linear regression with survey commands (i.e., prefix *svy* in Stata) accounting for longitudinal weights and clustering at the city level. Control variables included child’s age, sex, race/ethnicity, PA behavior at baseline, change in household income between T1 and T2, time elapsed between T1 and T2 interviews, as well as block group level change in median household income and total population between T1 and T2. The exposure variables were:

- Number of parks at T1.
- Change in the number of parks between T1 and T2.
- Upgrades to complete streets between T1 and T2.
- Number of upgrades (new and renovated opportunities) implemented between T1 and T2 in parks, PA facilities, bike lanes, and sidewalks.

All exposure variables were count variables measured at the 1-mile radius (around each child’s home) and with an exposure window of 18 months preceding T2. Because of the high correlation between upgrades to bike lanes, upgrades to sidewalks, and upgrades to complete streets, we created a scale including these variables, which we called street-related upgrades and used in the regression analysis instead of the three variables entered separately. A series of sensitivity analyses was run to ensure that results were robust to model specification and variable selection: (1) exposure variables upcoded to minimize the influence of any potential extreme values; (2) exposure window of 12 months, instead of 18 months, preceding T2; (3) exposure variables measured at the 0.5-mile radius instead of at the 1-mile radius; (4) subgroup analyses to assess whether the associations between the different elements of the PA environment and the outcome variable were different between (a) male vs. female children, and (b) younger (up to 12 years of age) vs. older (12+) children. Statistical significance was set at alpha = 0.05, based on two-tailed tests. Missing values were dropped and complete-case analyses were performed. All data management tasks and analyses were performed using Stata 17. [30].

## Results

The full longitudinal sample included 599 households, 548 of which had complete data for all variables used in the analysis. Of these 548, 81 were dropped because they moved (between T1 and T2) farther than 0.5 miles from their T1 residence and more than 6 months before T2. After exclusion of these cases, the analytic sample was comprised of 467 households (see Fig. 1).

Table 2 reports the characteristics of the analytic sample. Children’s mean age was 10 years, there was an equal split between males and females, and the most prevalent racial/ethnic groups were non-Hispanic Black (48%) and Hispanic (36%), followed by non-Hispanic White/other (16%). Children engaged in 60 min of PA at baseline for 3.8 days per week, on average. The change in PA between T1 and T2 was a normally distributed variable, based on visual inspection and on the skewness and kurtosis tests for normality (joint p-value = 0.183), with a mean of 0 and standard deviation of 3.1.

**Table 2:** New Jersey Child Health Study – Descriptive statistics of the analytic sample

	N or Mean	% or SD
Sex		
Females	229	49
Males	238	51
Race		
Hispanic	169	36
Non-Hispanic Black	225	48
Non-Hispanic White/Other	73	16
Age (Years)	9.7	3.3
Difference in family income^a^ between T1 and T2 (Income to poverty line ratio)	0.4	3.3
Difference in block group total population between T1 and T2	404	452
Difference in in block group household median income between T1 and T2 ($)	-8,588	10,590
Months between T1 and T2	38	18
PA at T1 (number of days per week)	3.8	2.5
Change in PA (number of days per week) between T1 and T2	0.0	3.1
Total	467	100

^a^Family income was calculated as the ratio between the annual income and the federal poverty line for the year of the survey and household size

Table 3 shows the average number of the PA environment variables used in the analysis. Most upgrades that occurred between T1 and T2 within a 1-mile radius of children’s homes were observed within the complete streets domain. Because there was a high correlation between the number of upgrades in complete streets and both (1) the number of upgrades in sidewalks (ρ = 0.70) and (2) the number of upgrades in bike lanes (ρ = 0.61), and because these three domains refer to upgrades that are related to streets, we combined them in a street-related upgrades scale (using the *alpha* command, with the *standardized* option), which had a Cronbach’s alpha value of 0.82. The command *alpha* creates a scale as the sum of the items included (here, the three types of street-related upgrades), divided by the number of items.

**Table 3:** New Jersey Child Health Study – Descriptive statistics of the exposure variables measuring elements of the PA^a^ environment within a 1-mile radius of respondents’ homes

	Mean	SD
Parks at T1	10.3	5.3
Change in the number of parks between T1 and T2	0.1	0.6
Parks upgrades	1.9	3.0
PA facility centers upgrades	0.2	0.8
Bike lanes upgrades	0.4	1.5
Complete streets upgrades	9.4	11.0
Sidewalks upgrades	1.0	1.1
Total	467	100

^a^Physical activity

Results from the linear regression analysis modeling the change in PA behavior between T1 and T2 are reported in Table 4. Most measures of the PA environment were not associated with the outcome, with one notable exception. The street-related upgrades scale was positively associated with the change in PA between T1 and T2; specifically, the change in PA was projected to be approximately 0.42 [(95% CI: 0.02, 0.82; p = 0.039) additional days for each one standard deviation increase in the street upgrade scale. Based on postestimation commands, when the street-related variable increases from 0 (its mean value) to 1 (one standard deviation), the expected change in PA increases from − 0.003 to 0.414, while holding all other variables at their means. PA behavior at baseline was inversely related to a change in PA between T1 and T2 as a result of a ceiling effect; children who were physically active for 5, 6, or 7 days per week did not have as much room for improvement as children who engaged in PA less often. However, on average, children who were more active at T1 also tended to be more active at T2, as the positive correlation between PA at T1 and PA at T2 (ρ = 0.22, p-value < 0.001) suggests. No other variables in the analysis were associated with the change in PA levels between T1 and T2.

**Table 4:** Results from survey-adjusted multivariable linear regression modeling the change in children’s PA^a^ behavior between T1 and T2

	Coefficient	95% CI	p-value
Age (Years)	0.00	(-0.12, 0.11)	0.956
Sex (ref: Males)			
Females	-0.02	(-0.68, 0.64)	0.943
Race (ref: non-Hispanic Black)			
Hispanic	-0.45	(-1.16, 0.26)	0.215
Non-Hispanic White/Other	-0.24	(-1.17, 0.69)	0.609
Difference in family income^b^ between T1 and T2	-0.01	(-0.06, 0.05)	0.792
Difference in block group total population between T1 and T2	0.00	(-0.03, 0.03)	0.934
Difference in in block group household median income between T1 and T2	-0.01	(-0.09, 0.08)	0.881
Months between T1 and T2	-0.02	(-0.04, 0.01)	0.144
PA at T1	-0.87	(-1.01, -0.74)	0.000
Parks at T1	-0.03	(-0.09, 0.03)	0.388
Change in the number of parks between T1 and T2	-0.16	(-0.73, 0.41)	0.576
Parks upgrades	-0.03	(-0.15, 0.09)	0.584
PA facility centers upgrades	-0.09	(-0.39, 0.22)	0.584
Street-related upgrades^c^	0.42	(0.02, 0.82)	0.039

^a^Physical activity

^b^Family income was calculated as the ratio between the annual income and the federal poverty line for the year of the survey and household size

^c^Street-related upgrades is a scale generated using the upgrades variables for (1) bike lanes, (2) complete streets, and (3) sidewalks

In the first set of sensitivity analyses, we replicated the model reported in Table 4 after recoding extreme values for all the PA environment variables; specifically, values greater than the mean plus 3 standard deviations were upcoded to avoid the influence of these extreme values. Results were almost identical to those presented in Table 4. Second, we used 12 months instead of 18 months as the period over which the T2 values for the PA environment variables were calculated. These results were largely the same as those from the main model. Third, we used the PA variables capturing changes in the environment within a 0.5-mile (instead of 1-mile) radius from respondents’ homes. The coefficient for the street upgrade scale in this model was the same in terms of direction and similar in magnitude but was not significant—most likely because of the reduced variability of the PA variables within a 0.5-mile radius. Lastly, subgroup analyses showed that the associations observed in the main model, between the different elements of the PA environment and the change in PA levels between T1 and T2, were not different across sex or age groups. The association between street-related upgrades and change in PA was of greater magnitude for females and for older children, compared to males and younger children, respectively. These differences, however, were not statistically significant.

## Discussion

Street-related upgrades within a mile of children’s homes were independently and significantly associated with an increase in children’s PA; subgroup analyses revealed that this association did not differ across sex or age groups. The street-related upgrades included complete street, sidewalk, and bicycle lane upgrades. In contrast, changes to the number of parks between T1 and T2, and upgrades to parks and PA facilities were not independently associated with a change in children’s PA from T1 to T2. Individual and family level variables were also not associated with the change in PA levels. While previous studies have shown that PA tends to decline in children 10 years of age or older, [31, 32] in our sample such an association was not observed.

It is notable that our study, one of few to benefit from the added strengths associated with using a longitudinal design, found significant effects of relatively low-cost interventions—often considered in community interventions but rarely studied for evidence of their impact on prevention. Studies examining the association between bicycle lanes and children’s PA find similar results to those of the current analysis. A systematic review and meta-analysis of 20 recent studies shows a strong positive association between bike lane access and children’s PA. [5] The only longitudinal study included in the review was conducted in Seattle, where sidewalk modifications and bike lane accommodations near schools have increased since 2007. [33] Increased bike lane accommodations (e.g., adding cycle tracks, separated/buffered bike lanes) near schools was correlated with more students bicycling to school. Similarly, an increase in sidewalks over time was associated with an increase in students walking to school. Greater access to foot and bike paths within 300 m of home has also been found to be associated with greater amounts of PA. [34].

Cross-sectional studies examining the association between different elements of the PA environment and PA behaviors among children have shown mixed results. For instance, An et al., using data from the Centers for Disease Control and Prevention’s cross-sectional National Survey of Children’s Health, observed a negative association between neighborhood availability of sidewalks and levels of PA and a positive association between availability of parks and playgrounds and levels of PA among children with special healthcare needs. [35] Neighborhood availability of a recreation center was associated with higher PA among children without special healthcare needs. [35] A different conclusion was reached from a study on Swedish children, in which counts of recreational areas such as playgrounds and open green spaces were not associated with PA. [34] Goon at al. observed an association between sidewalks and children’s PA; specifically, the absence of sidewalks in the neighborhood was associated with 2.6 fewer minutes per day of moderate to vigorous PA and with 7 more minutes per day of sedentary time among 9-14-year-old Canadian children. [36].

Results from cross-sectional studies examining associations between parks and children’s PA have also been mixed, with some observing increased PA with higher park density or proximity, [36–39] and others finding no association. [40–46] The current analysis did not show associations between changes in the number of parks and PA behaviors; however, this null result might derive from the limited change in the number of parks over the follow-up period.

Renovated opportunities in parks in our study were also not associated with children’s PA. Cohen et al. did observe an increase in PA by both children and adults in San Francisco parks that had been extensively renovated, compared to parks that had not undergone renovation. [47] The nature of park renovations observed in our study was not extensive, but rather was confined to moderate improvements to existing structures, such as resurfacing of a basketball court.

Many population-based intervention efforts to promote active living are focused on implementing incremental changes or upgrades to existing facilities and improvements in existing infrastructure. The data collection protocol we employed is designed to document new opportunities, upgrades, and renovations within existing facilities at a granular level that is generally not available for large-scale studies. Publicly available databases can provide signals of change; however, they are often compiled for other purposes and may not provide adequate detail for research. For example, parks data available from municipalities may not capture upgrades to park features or creation of new opportunities for physical activity within existing parks.

Our study focused on these smaller, or microscale, changes to the built environment since they are more common and are easier to implement than macroscale changes. While the current study is specific to four urban cities in New Jersey that underwent changes as part of several local, state, and federal initiatives, the study protocol can be applied to other geographic regions with diverse populations and varied interventions. Locally relevant key informants (e.g., for identifying intentional interventions) can be found through local chapters of the American Public Health Association (APHA), or Society for Public Health Education (SOPHE), related advocacy or non-profit organizations, YMCAs, state university and local extension offices, public health officers and officials, municipal and county departments of public works and engineering, and city planners.

Among the strengths of the current study is the use of a systematic protocol to documented incremental changes to existing PA opportunities. This protocol, developed in collaboration with city planners and PA experts, was designed to include all PA upgrades that were actually implemented (and not just planned). Other strengths included the longitudinal design, which allowed us to examine individual changes over time, and the detailed geographic information that was collected, which allowed us to determine which PA upgrades occurred around the home of every household in the sample. The current study also had some limitations. For instance, despite the level of detail of the data collection protocol, given the breadth of potential change that it is designed to monitor, it is possible that some changes to the PA environment were not detected. It is also possible that micro-scale aspects of the PA environment may have deteriorated or ceased to exist during the study period, but ongoing monitoring of such existing features was beyond the capacity of the study. Strengthening our ability to detect purposeful, intended changes, multiple sources of data were integrated to ensure that the vast majority of upgrades that occurred in these communities over the study period were captured, improving upon strategies used in previous studies. However, most data sources we used were designed to serve non-research purposes (e.g., project specification and construction plans for PA changes). As a corrective, two analysts independently coded the exposure variables, and extreme care was taken to confirm the accuracy of the information incorporated in these variables; nevertheless, errors may still have occurred. Additionally, the level of PA for children was assessed through parent reports, which is less precise than objective measures (i.e., through accelerometry data), especially for older children, as parents may not monitor them as closely. This measurement issue is likely to have added random noise to the estimates, as it would have occurred for all children, regardless of their proximity to an upgrade. Further, while for each child in the sample we were able to measure their neighborhood PA environment at baseline and the changes to that environment that occurred during the follow-up period, we do not have information on whether and to what extent children actually used the different elements of their PA environment. With 467 respondents in the analytic sample, subgroup analyses might have been underpowered. Lastly, the study is representative of four predominantly low-income, densely populated cities in New Jersey; thus, results might not be generalizable to different populations or contexts.

## Conclusion

This study provides evidence of the value to public health of relatively low-cost interventions, the implementation of which is within reach of local policymakers and community organizations. Using a detailed protocol that identified both micro- and macro-scale changes in the PA environment and a longitudinal study design, we found that street-related upgrades, specifically complete street, sidewalk, and bicycle lane upgrades, were associated with an increase in children’s PA. The current study supports funding of projects that aim at improving streets and sidewalks in cities.

## Data Availability

The datasets analyzed during the current study will be made available from the corresponding author upon reasonable request.

## Abbreviations

PA: Physical Activity
T1: Time 1
T2: Time 2
NJCHS: New Jersey Child Health Study
CDC: Centers for Disease Control and Prevention
OPRA: Open Public Records Act
CDO: Community Development Organizations
